# Colonic Metastasis with Anemia Leading to a Diagnosis of Primary Lung Adenocarcinoma

**DOI:** 10.1155/2016/5275043

**Published:** 2016-01-27

**Authors:** Vasa Jevremovic, Amer Abboud, Stuart Krauss

**Affiliations:** ^1^Department of Oncology, Weiss Memorial Hospital, Chicago, IL 60640, USA; ^2^Department of Pathology, Weiss Memorial Hospital, Chicago, IL 60640, USA

## Abstract

Metastasis occurs with 50% of lung carcinomas, most commonly to lymph nodes, adrenal glands, liver, bone, and brain. It is extremely rare for lung cancer to present with symptoms of a gastrointestinal metastasis and even more so pertaining to the colon. To the best of our knowledge, only 12 such cases have been reported in the literature. We describe a case of a 71-year-old female presenting with refractory iron deficiency anemia that was found to have a lesion in the transverse colon. Pathology revealed adenocarcinoma of the lung and a subsequent lung lesion was discovered in a retrograde fashion.

## 1. Introduction

Lung cancer is a common malignancy with roughly 50% of cases demonstrating metastasis [1]. The most commonly affected sites for metastasis are lymph nodes, adrenal glands, liver, bone, and brain [1–3]. Several autopsy studies have shown metastasis to the gastrointestinal (GI) tract more commonly than previously thought, occurring in approximately 0.2% to 11.9% of cases but usually with diffuse metastatic disease [2]. Symptomatic GI metastasis of lung cancer is extremely rare, estimated to occur in 0.2% to 0.5% of cases [2, 4, 5]. It is possible that GI metastases from lung carcinoma are being underdiagnosed due to attribution of symptoms to effects of chemotherapy, such as ulceration, enteritis, or colitis [2]. Regardless, the prevalence of symptomatic colonic metastasis from a primary lung carcinoma is an uncommon event. In this report, we describe a primary lung adenocarcinoma presenting with a colon lesion and subsequent anemia.

## 2. Case Presentation

A 71-year-old female with a history significant for type II diabetes mellitus, hypertension, cholelithiasis, hyperlipidemia, hemorrhoids, and chronic kidney disease presented to clinic for treatment of anemia with an iron deficiency clinical picture. Treatment began with orally administered iron supplementation, which was not tolerated due to gastrointestinal symptoms. The patient was initiated on intravenous iron supplementation on a weekly basis with monitoring of hemoglobin and iron studies. Hemoglobin and ferritin levels marginally improved. The patient had tobacco use of 110 pack-years. Family history was significant for coronary artery disease in the patient's father, and breast carcinoma in two paternal aunts. Review of systems showed that the patient was experiencing fatigue, but was unremarkable otherwise. This included other signs of anemia such as tachycardia, syncopal symptoms, and presence of hematochezia.

Physical exam was unremarkable with the exception of a significantly positive fecal occult blood test. The patient was thus referred to the gastroenterologist for endoscopy studies to investigate the source of GI bleeding as the cause of anemia.

**Figure 1:**
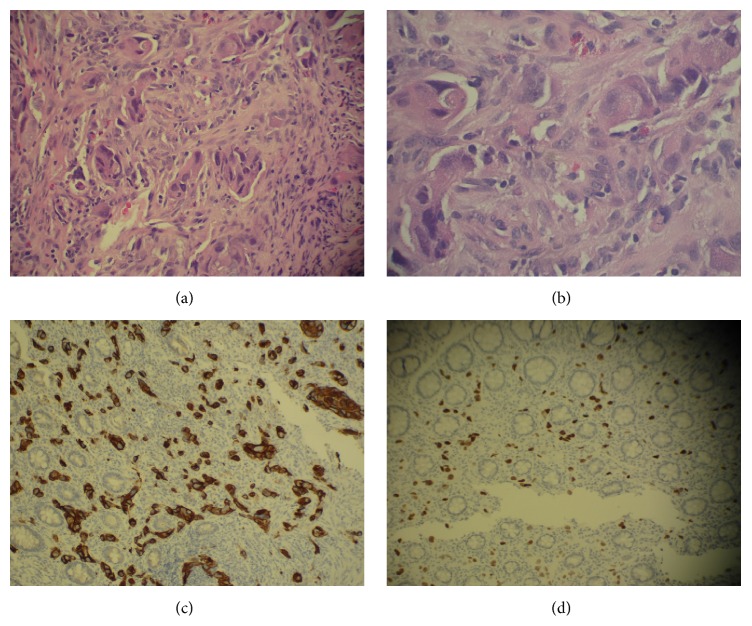
Staining of transverse colon biopsy. (a) H&E stain of transverse colon biopsy. Infiltrating poorly differentiated adenocarcinoma between normal colon glands. (b) H&E stain of biopsy from colon demonstrating poorly differentiated glandular structure. (c) Strong positive reaction for CK7 immunostaining. (d) Adenocarcinoma with positive reaction for TTF-1.

Upper and lower endoscopies of the GI tract demonstrated hyperplastic polyps in the stomach, rectum, cecum, and transverse colon, chronic inactive gastritis, and absence of* Helicobacter pylori*, as well as a lesion in the transverse colon appearing as a hyperplastic polyp. All lesions were less than one centimeter in size. Immunohistochemistry of the mass in the transverse colon exhibited negative reaction for S100, melanin-A, c-kit, dog-1, CD34, estrogen receptor, CK20, CA19-9, and CA125. The battery of immunohistochemistry was positive for cytokeratin AE1/AE3, TTF-1, and CK7 (Figure 1). This constellation favors a tissue diagnosis of lung adenocarcinoma.

**Figure 2:**
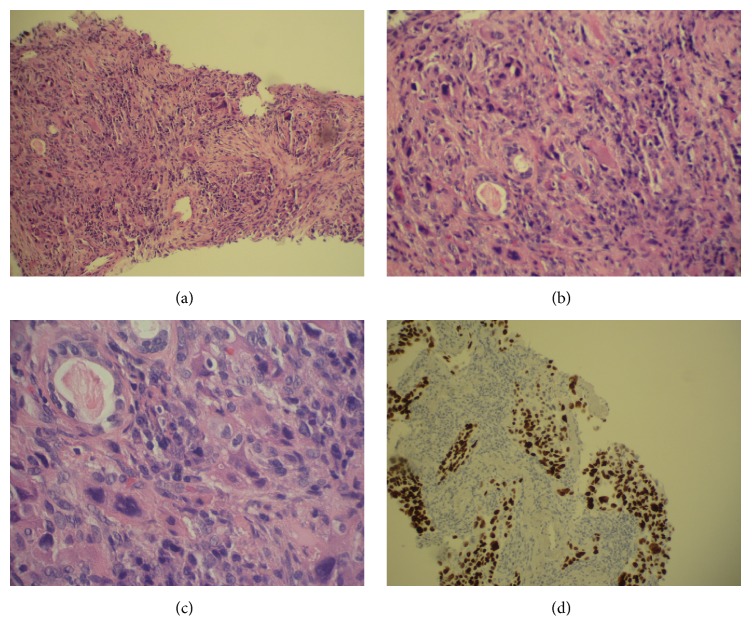
Needle core biopsy of left upper lung lobe mass. (a), (b), and (c) H&E stain demonstrating infiltrating adenocarcinoma. (d) Immunohistochemistry positive reaction for TTF-1, consistent with primary lung malignancy and the metastatic lesion in the transverse colon.

**Figure 3:**
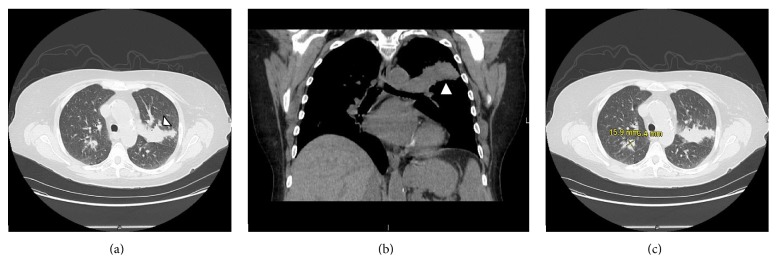
CT scan of the chest demonstrating lung lesions. (a) Transverse view CT scan without IV contrast shows a 3.6 by 4.9 by 2.9 cm mass in left upper lobe* (arrow)*. Interaction with the oblique fissure is noted. (b) Coronal view showing left upper lobe lesion* (arrow)* without any mediastinal or hilar lymphadenopathy. (c) A pulmonary nodule is visualized in the right lung (contralateral). This mass was not biopsied and was assumed to be secondary to the primary adenocarcinoma in the left lung.

Computed tomography (CT) of the chest, abdomen, and pelvis with oral contrast was scheduled in order to stage the patient's malignancy. The day before the CT scan was to take place, routine blood work revealed an acute drop in hemoglobin to 6.5 g/dL and she was admitted to the hospital. As an inpatient, two units of packed red blood cells were transfused, stabilizing her hemoglobin at 8.5 g/dL, and the CT scan was pursued. Intravenous contrast was not used due to the patient's elevated creatinine of 1.80 mg/dL. The scan showed a 3.6 by 4.9 by 2.9 cm mass in the posterior left upper lobe abutting the oblique fissure with two additional pulmonary nodules and no evidence of mediastinal or hilar lymphadenopathy (Figure 3). One of the pulmonary nodules was located in the contralateral lung (Figure 3(c)). No masses were appreciated in the abdomen and colonic diverticulosis was the only disease process identified in this region. Percutaneous biopsy of the left lung mass with CT guidance was undertaken the next day. The needle core biopsy demonstrated adenocarcinoma of the lung, with positive staining for TTF-1 (Figure 2). The final diagnosis was a primary lung adenocarcinoma with distant metastases to the contralateral lung, ipsilateral lung, and transverse colon.

## 3. Discussion

Gastrointestinal metastasis of primary lung carcinoma is uncommon, yet it is thought to be underdiagnosed [2]. The small bowel is an unusual location for lung carcinoma to metastasize to and is the most common site within the GI tract; the colon is an even more unusual destination for such metastatic disease [6]. Clinical prevalence of GI metastasis seems to be much lower than those evident at autopsy [7]. To the best of our knowledge, of the 12 clinical cases of lung cancer metastasis to the colon reported in literature [1, 8–12], 10 had exhibited a tissue diagnosis of squamous cell carcinoma [2]. One case report describes a metastatic lung adenocarcinoma to the colon mimicking a colonic polyp [6] similar to this case report.

The correct identification of a gastrointestinal metastasis is difficult to ascertain, as either CT scan or endoscopy does not demonstrate unique features distinguishing the lesion as a primary lung malignancy [13]. Therefore, diagnosis is largely reliant on immunohistochemical staining; utilization of positive TTF-1, CK7, and CK20 as markers for diagnosis of primary lung carcinoma is essential [2, 13]. For our patient, the lesion was immunoreactive for TTF-1, CK7, and cytokeratin AE1/AE3, favoring the diagnosis of adenocarcinoma of the lung. Negative staining with regard to p63 rules out undifferentiated squamous cell carcinoma of the lung.

Considering that metastasis from the lung to the colon is thought to be clinically asymptomatic in the majority of cases, fecal occult blood testing is a useful tool in detection [2]. This may reveal GI bleeding of various grades ranging from anemia and melena to GI bleeds requiring immediate intervention [6]. Other symptoms of colonic metastasis may include bowel obstruction or bowel perforation secondary to tumor erosion [6]. In our case, the patient had not experienced respiratory symptoms and was unremarkable on physical examination despite the considerable size of the primary lesion. Thus, diagnosis of lung carcinoma was made on the basis of anemia secondary to colonic metastasis. In one case report, primary lung carcinoma with colonic metastasis was diagnosed with the use of positron emission tomography (PET) scan [2, 14]. The primary lung lesion was known, in which PET scanning was employed for disease staging revealing a colonic lesion. However, PET scan is not a sensitive modality for investigating GI metastasis of lung cancer as it detects extrathoracic metastasis in approximately 25% of patients [9]. Prognosis of lung cancer seems to be worse when complicated with GI metastasis, mainly due to acute bleeding and perforation associated with chemotherapy [7]. This is evident with the case at hand, in which an acute decrease in hemoglobin required hospitalization.

In conclusion, symptomatic metastasis to the colon from a primary lung carcinoma is highly unusual. Detection is often incidental on staging work-up for a primary lung mass, yet it can lead to a diagnosis of a primary lung lesion in retrograde fashion in the absence of respiratory symptoms.
